# Comment on Propofol and survival: an updated meta-analysis of randomized clinical trials

**DOI:** 10.1186/s13054-023-04550-2

**Published:** 2023-07-11

**Authors:** Adam Glass, Philip McCall, Ben Shelley

**Affiliations:** 1 Wellcome-Wolfson Institute for Experimental Medicine, Queen’s University Belfast, Belfast, UK; 2 Academic Unit of Anaesthesia, Pain and Critical Care, University of Glasgow, Glasgow, UK

Dear Editor,

We wish to add further comment to the recent publication by Kotani et al. [1]. Firstly, we would like to thank the authors for undertaking a thorough and detailed meta-analysis on a clinically important topic suggesting propofol is associated with increased mortality (RR 1.10, 95% CI 1.01–1.20, *p* = 0.03). We would, however, like to add to the comments of Benavides-Zora et al.[2] and Hansel [3] regarding the meta-analysis.

## Mortality outlier study

As already highlighted by Benavides-Zora et al. [2] and Hansel [3], there is an error in the data extraction regarding the mortality rate from the manuscript by Likhvantsev et al. [4]. The corrected one-year mortality rate in this study is 52/292 (17.8%) in the sevoflurane group and 81/326 (24.8%) in the propofol group, rather than 52/450 (11.6%) and 81/450 (18%), respectively, as detailed in the Kotani et al. meta-analysis. As detailed by Hansel, this artificially inflates the estimated risk ratio of the study in the meta-analysis.

On closer analysis of the Likhvantsev et al. study, the mortality rate in both groups is very high and a significant outlier in the meta-analysis. The study included 900 patients undergoing elective coronary artery bypass grafting (CABG) with “*patients who had a recent or ongoing myocardial infarction*” excluded. The study authors comment that their “*1-year mortality was extremely high in this cohort of patients*” and their “*hypotheses to explain these findings was that patients’ adherence to cardiologic medication was extremely poor after hospital discharge*”. In comparison, the crude one-year mortality for “all cardiac surgery” in the UK is 3.78% [5]. This study is one of only two out of 252 studies included in the meta-analysis demonstrating increased mortality in a propofol group. With such a markedly high mortality rate, this study is a clear outlier and has the potential to influence the meta-analysis results, in addition to the data extraction error.

## Study not included in the analysis

Further investigation of the 900-patient study by Likhvantsev et al. [4] shows that two authors (Likhvantsev and Landoni) went on to perform a 5400-patient randomized control trial comparing maintenance of anaesthesia in patients undergoing elective CABG with volatile anaesthetic (2709 patients) to total intravenous anaesthetic (TIVA, 2691 patients) [6]. This later paper demonstrated no significant mortality difference at 1 year (relative risk, 0.94; 95% [CI] 0.69 to 1.29; *p* = 0.71). Mortality rates in this study were 2.8% in the volatile group and 3.0% in the TIVA group, markedly different to the 17.8% and 24.8% reported in the previous 900 patient study of Likhvantsev et al. and more in keeping with contemporary reports [4].

We must highlight that whilst the 900-patient study of Likhvantsev et al. [4] is included in the meta-analysis, the 5,400-patient study of Landoni et al. [6] is not. This may be due to the concise literature search strategy used in the meta-analysis in which the Landoni et al. paper does not appear as the intervention was documented as TIVA and not propofol. In the TIVA group, however, anaesthetic was maintained by a propofol infusion in 2297/2665 (86.9%) patients allowing comparison against 2709 patients with volatile anaesthetic maintenance. It is therefore surprising that this study was not included in the meta-analysis given; the detailed data collection methods described, it is mentioned in the discussion section and, most surprisingly, that the first author (Landoni) is a co-author in this meta-analysis [1].

## Spin

Again, we wish to add to the comment of Benavides-Zora et al. [2] in regard to the “spin” of the presentation of results. In Kotani et al.’s discussion of the difference between the meta-analysis and the 5400 patient Landoni et al. study, they comment, “*We hypothesize that the use of propofol in the majority of patients who were randomized to the volatile group* [crossover between treatment groups] *blunted the detrimental effect of propofol on survival in this trial*”. This contradicts a sub-group analysis in their paper that showed no difference in mortality between groups in studies where propofol was not used in the comparator arm (RR 1.03, 95% CI 0.94–1.14, *p* = 0.50).

As Hansel commented, Kotani et al. “*stop short of addressing the elephant in the room*” of the impact of the 900 patient Likhvantsev et al. on the overall meta-analysis results. In addition, we fell that they fail to address the white elephant in the room of the 5400-patient study of Landoni et al. which mysteriously does not feature in the meta-analysis at all.

## Summary

In summary, we believe that the readers of Critical Care should be cautious in their interpretation of the results of this important meta-analysis and would no doubt appreciate re-analysis without the significantly outlier in mortality of the Likhvantsev et al. study and including the mortality data from the 5400-patient Landoni et al. study.

## Data Availability

Not applicable.
